# Improved Metastatic-Free Survival after Systematic Re-Excision Following Complete Macroscopic Unplanned Excision of Limb or Trunk Soft Tissue Sarcoma

**DOI:** 10.3390/cancers16071365

**Published:** 2024-03-30

**Authors:** Francois Gouin, Audrey Michot, Mehrdad Jafari, Charles Honoré, Jean Camille Mattei, Alexandre Rochwerger, Mickael Ropars, Dimitri Tzanis, Philippe Anract, Sébastien Carrere, Dimitri Gangloff, Agnès Ducoulombier, Céleste Lebbe, Jérôme Guiramand, Denis Waast, Frédéric Marchal, François Sirveaux, Sylvain Causeret, Pierre Gimbergues, Fabrice Fiorenza, Brice Paquette, Pauline Soibinet, Jean-Marc Guilloit, Louis R. Le Nail, Franck Dujardin, David Brinkert, Claire Chemin-Airiau, Magali Morelle, Pierre Meeus, Marie Karanian, François Le Loarer, Gualter Vaz, Jean-Yves Blay

**Affiliations:** 1 Surgery Department, Centre Léon Bérard, 69008 Lyon, Francegualter.vaz@lyon.unicancer.fr (G.V.); 2 Surgery Department, Institut Bergonié, 33076 Bordeaux, France; 3 General and Digestive Oncologic Surgery, Centre Oscar Lambret, 59000 Lille, France; 4 Surgery Department, Gustave Roussy Cancer Campus, 94805 Villejuif, France; 5 Orthopedic and Traumatologic Surgery Department, Hôpital Nord, Hopital de la Conception, APHM, 13005 Marseille, France; 6 Orthopedic Surgery Department, CHU de Rennes, 35033 Rennes, France; mickael.ropars@chu-rennes.fr; 7 Surgery Department, Institut Curie, PSL University, 75248 Paris, France; dimitri.tzanis@curie.fr; 8 Orthopedic Surgery Department, Hôpital Cochin, AP-HP, Université de Paris, Faculté de Santé, Unités de Formation et de Recherche de Médecine, 75015 Paris, France; 9 Surgery Department, Institut de Recherche en Cancérologie, 34298 Montpellier, France; 10 Surgery Department, Toulouse Oncopole, 31100 Toulouse, France; 11 Senology Surgery Department, Onco-Gynécologique et Reconstructrice, Centre Antoine Lacassagne, 06100 Nice, France; 12 Reconstructive et Esthetic Plastic Surgery, Hôpital Saint Louis, 75010 Paris, France; 13 Surgery Department, Institut Paoli Calmette, 13009 Marseille, France; 14 Orthopedic and Traumatologic Surgery Clinic, CHU Nantes, 44093 Nantes, France; 15 Surgery Department, Institut de Cancérologie de Lorraine, Université de Lorraine, CNRS, CRAN, UMR 7039, 54000 Nancy, France; 16 Orthopedy Department, CHU de Nancy, 54000 Nancy, France; 17 Surgery Department, Centre George-François Leclerc, 21079 Dijon, France; scauseret@cgfl.fr; 18 Surgery Department, Centre Jean Perrin, 63011 Clermont Ferrand, France; 19 Orthopedic and Traumatology Surgery Department, CHU Limoges, 87000 Limoges, France; 20 Department of Digestive Surgery, Jean Minjoz University Hospital, 25000 Besançon, France; bpaquette@chu-besancon.fr; 21 Medical Oncology Department, Institut Godinot, 51100 Reims, France; 22 Visceral et Digestive Surgery Department, Centre François Baclesse, 14076 Caen, France; 23 Onco-Orthopedic Surgery Department, Hôpital Trousseau, CHRU de Tours, 37000 Tours, France; 24 Medical Oncology and Surgical Oncology Department, Centre Henri Becquerel, 76038 Rouen, France; 25 Orthopedic Surgery Department, CHU de Strasbourg, 67200 Strasbourg, France; david.brinkert@chru-strasbourg.fr; 26 Clinical Research and Innovation Department, Centre Léon Bérard, 69008 Lyon, France; 27 Department of Biopathology, Centre Léon Bérard, 69008 Lyon, France; 28 Anatomo-Pathology Surgery Department, Institut Bergonié, 33076 Bordeaux, France; 29 Department of Medical Oncology, University Claude Bernard Lyon I, 69008 Lyon, France; 30 Department of Medicine, Léon Bérard Center, Unicancer, 69008 Lyon, France

**Keywords:** soft tissue sarcoma, surgery, reference center, multidisciplinary tumor board, resection margins, metastatic free survival

## Abstract

**Simple Summary:**

The quality of resection after unplanned excision of soft tissue sarcoma (STS) performed outside of a reference center or at second resection potentially impacts local and metastatic recurrence and survival. The French cohort NETSARC prospectively collected data from patients with unplanned excision outside reference centers from 2010 to 2019 and reported survival in patients reexcised (RE) or not (No-RE). Patients who would most benefit from RE need to be identified. A total of 2371 patients had unplanned excision for STS outside reference centers, including 1692 patients with no multidisciplinary board review (RE: 913; No-RE: 779). Discrepancies in RE/No-RE subgroups were observed regarding age, tumor site, size, depth, grade and histotype. R0 final resection associated with better MFS; R1 initial resection showed better MFS than R0 initial resection. The study identified RE as an independent favorable factor for MFS (HR 0.7, 95% CI 0.53–0.93; *p* = 0.013). All subgroups except patients > 70 years, and patients with large tumors (>10 cm) showed better MFS with RE. RE in patients with STS of limb or trunk after macroscopic complete resection out of NETSARC reference center, and also in R0 resections to improve LRFS and MFS. Systematic RE should not be advocated for patients ≥ 70 years, or tumor size ≥ 10 cm.

**Abstract:**

Background: Whether re-excision (RE) of a soft tissue sarcoma (STS) of limb or trunk should be systematized as adjuvant care and if it would improve metastatic free survival (MFS) are still debated. The impact of resection margins after unplanned macroscopically complete excision (UE) performed out of a NETSARC reference center or after second resection was further investigated. Methods: This large nationwide series used data from patients having experienced UE outside of a reference center from 2010 to 2019, collected in a French nationwide exhaustive prospective cohort NETSARC. Patient characteristics and survival distributions in patients reexcised (RE) or not (No-RE) are reported. Multivariate Cox proportional hazard model was conducted to adjust for classical prognosis factors. Subgroup analysis were performed to identify which patients may benefit from RE. Results: Out of 2371 patients with UE for STS performed outside NETSARC reference centers, 1692 patients were not reviewed by multidisciplinary board before treatment decision and had a second operation documented. Among them, 913 patients experienced re-excision, and 779 were not re-excised. Characteristics were significantly different regarding patient age, tumor site, size, depth, grade and histotype in patients re-excised (RE) or not (No-RE). In univariate analysis, final R0 margins are associated with a better MFS, patients with R1 margins documented at first surgery had a better MFS as compared to patients with first R0 resection. The study identified RE as an independent favorable factor for MFS (HR 0.7, 95% CI 0.53–0.93; *p* = 0.013). All subgroups except older patients (>70 years) and patients with large tumors (>10 cm) had superior MFS with RE. Conclusions: RE might be considered in patients with STS of limb or trunk, with UE with macroscopic complete resection performed out of a reference center, and also in originally defined R0 margin resections, to improve LRFS and MFS. Systematic RE should not be advocated for patients older than 70 years, or with tumors greater than 10 cm.

## 1. Introduction

Soft tissue sarcomas (STS) are a heterogenous group of malignant tumors gathering over 155 histotypes and molecular subtypes that constitute approximatively 1% of all malignancies [1]. Extremities and trunk-wall locations are the most frequent location of STS [2]. En-bloc surgical resection with clear margins (R0) after review by a multidisciplinary tumor board (MDTB) in an expert center is the mainstay of curative treatment [3].

Nevertheless, unplanned excisions (UE) outside of a reference center occurred regularly, and pre-operative imaging, accurate diagnosis, and discussion in a multidisciplinary tumor board to guide treatment strategy are not systematically performed [4,5,6,7,8]. Patients referred after UE range from 12% to 71% [4,5,6,7,8].

In case of macroscopically complete UE outside of a specialized center, re-excision (RE) followed by radiotherapy is generally considered [3,9,10]. Most studies reported that RE improved local control [5,11,12,13,14,15,16,17,18,19,20]. The impact of RE on metastatic-free survival (MFS) is still debated [12,21,22], raising the issue of the most appropriate management, based on surveillance measures exclusively or considering a more aggressive approach with systematic RE.

The presence of residual tumor in tumor beds has been reported to be associated with oncologic outcome, and several studies reported that patients benefit from RE after UE [5,6,17,20,23,24,25,26,27] while others did not evidence a correlation between residual tumor in tumor bed and improved distant metastasis risk and overall survival (OS) [28], or disease specific survival [22], and reported similar OS in patients with initial UE whether patients were re-operated or not [12].

The present study reports on the impact of systematic RE in patients with STS of limb or trunk, prospectively registered in the French nationwide database NETSARC+, with STS UE performed out of a reference center, qualified as macroscopically complete. We explored the impact of margin status on MFS.

## 2. Patients and Methods

### 2.1. NETSARC Network and Database

The French nationwide reference network for clinical and pathological sarcoma care NETSARC supported by the French Institute of Cancer (INCa) set up a nationwide database of all STS diagnosed in France, currently considered to be close to exhaustivity [2]. All sarcomas including suspicion for sarcoma are presented and reviewed by a multidisciplinary tumor board (MDTB) involving experts from the 26 French cancer centers, and are registered in a database by a dedicated team of clinical research assistants, at first presentation, at any time of the disease course (before diagnosis, before any treatment, after primary surgery, before adjuvant therapy, at the date of oncologic event or/and clinical trial screening).

In France, each operated STS patient regardless of the institution should benefit from a centralized review with double-interpretation and pathological reports encourage the clinicians to present each case to MDTB. Thus, data from all patients whether or not they were operated in or outside NETSARC+ reference center network are collected in NETSARC+ database.

The database includes patient and tumor characteristics, surgery, relapse and survival. The wider tumor diameter defined tumor size. The National Federation of Cancer Centres Unicancer categorized histological grades as grade 1, 2, 3, and ungraded tumors resulting from histology grading failure or lack of critical elements to complete the diagnosis, according to experts.

**Figure 1:**
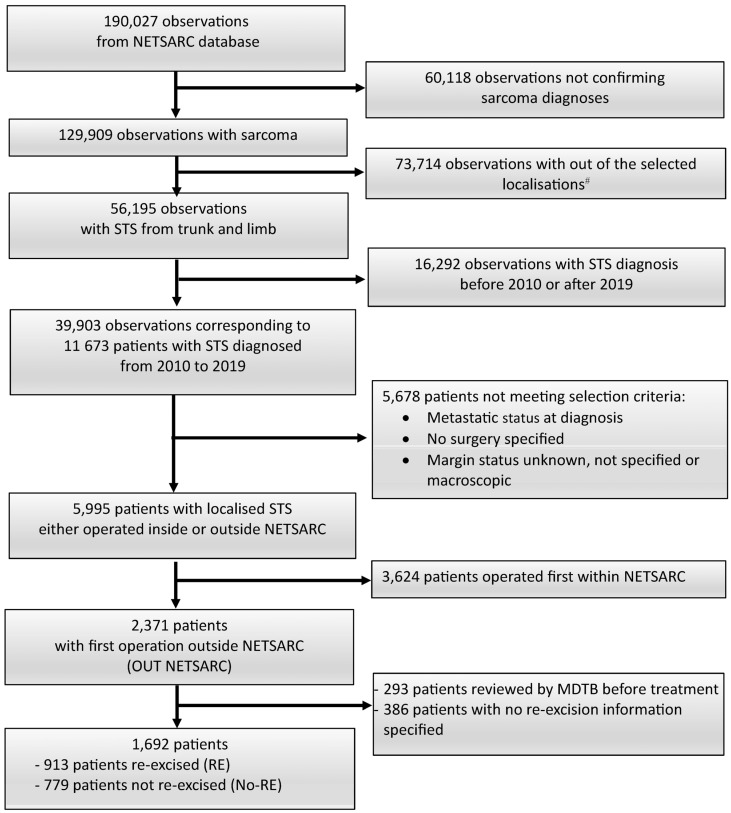
Flow-chart. The statistical unit is successive observations reviewed by the MDTB, and patients; # bone (*n* = 23,452 observations); viscera (*n* = 19,249); head and neck (*n* = 5968); internal trunk (*n* = 24,678); soft tissue (*n* = 338); unknown (*n* = 29). MDTB: multi-disciplinary tumor board.

The quality of surgical resection was defined according to the definition of the Union Internationale Contre le Cancer, UICC [29]. R0 indicated clear margins. The present study qualified R0 margins for a monobloc resection with clear margins specified on pathological report; R1 margins referred to potential microscopic residual disease i.e., visible tumor cells on resection margins (positive microscopic margins). The initial margins referred to margins initially documented using pathology and surgery reports when available; R1 margins indicated margins not confirmed as R0 or R2 based on pathologic and surgical report. R2 resulted from fragmented resections, or operative/pathological reports suggesting or notifying macroscopic residual tumor and/or fragmented resection were excluded as cases with no margin characterization (Figure 1). Each case presented after the first surgery required MDTB decision to re-operate the patient based on patient general assessment, post-MRI, pathologic and operative features provided in reports, when available.

### 2.2. Patient Selection

The study analyzed data from patients with localized STS of trunk and limbs diagnosed between 2010 and 2019, registered in NETSARC database. Patients experienced initial surgery outside a NETSARC reference center. Desmoid, well differentiated (atypical lipomatous tumors), dermato-fibrosarcoma protuberans were excluded. Patients with metastasis at diagnosis or unknown initial metastatic status were also excluded. Patients operated outside a NETSARC center, with prior MDTB assessment before surgery, were excluded and considered as having experienced a planned resection out of a reference center (Figure 1).

The affiliation of the first surgeon was used to categorize patients as treated within or outside a NETSARC reference center; patients were considered as operated in a NETSARC reference center if the surgeon was registered in NETSARC network (https://NetSarc.sarcomabcb.org, accessed on 9 November 2022), and conversely, as operated in a non-expert center if the surgeon was not referenced in the NETSARC network.

### 2.3. Objectives

This study aims at assessing whether re-excision (RE) and margins assessed at first and last surgery impacted Metastasis-Free Survival (MFS) in patients with STS of limbs or trunk, who had experienced unplanned excision (UE), qualified as macroscopically complete, outside of NETSARC reference centers. A sub-group analysis was conducted to identify subgroup of patients benefiting from RE. Secondary objectives explored the impact of final margin status and initial margin status—as documented at initial surgery outside of NETSARC centers—on MFS, local relapse free survival (LRFS) and OS in patients with or without systematic RE.

The study received approval from the national Advisory Committee on Information Processing in Health Research (Comité consultatif sur le traitement de l’information en matière de recherche dans le domaine de la santé, CCTIRS) n° 10.403, 16 September 2010, and from the French data protection authority (Commission Nationale Informatique et Liberté, CNIL), n° 910,390, 15 July 2013.

### 2.4. Statistical Methods

Qualitative variables were described with frequencies and percentages, and quantitative variables with average and range. Comparisons between groups used the chi-square test for qualitative variables and Kruskall–Wallis test for quantitative variables. The diagnosis date was the date of pathological diagnosis (biopsy or first surgery). Metastasis-Free Survival (MFS) was defined as the time from the date of diagnosis to the date of the last follow-up or the date of metastatic progression. MFS was estimated using the Kaplan–Meier method, and duration of follow-up was determined using the reverse Kaplan–Meier method and expressed with Q1–Q3 interval. MFS distributions were compared between groups using the Log–rank test. Univariate and multivariate regressions were performed using the Cox proportional hazard model, and included usual prognostic factors for sarcoma. We explored the impact of systematic re-excision (RE) as adjuvant care on survival; To take into account the correlation between the quality of initial and final surgery, two distinct multivariate models were used considering each of the two variables (one model related to the initial margins/quality of initial resection, one model related to final margins/quality of final resection). Subgroup analyses explored whether RE may benefit to specific subgroups of patients. The cut-off date for data analysis was 9 November 2022. Analyses were performed using SAS version 9.4 software (SAS Institute Inc., Cary, NC, USA) and significance for all statistical tests was evaluated using two-sided *p* values.

## 3. Results

### 3.1. Patient and Tumor Characteristics

Among the 1692 patients operated outside NETSARC centers, with microscopic margins specified at initial UE surgery and re-excision information available, 913 patients were re-excised (RE) and 779 patients had no re-excision (No-RE) (Figure 1).

The median follow-up was 31.4 (Q1–Q3: 6.7–61.6) months.

Patient characteristics are reported on Table 1. Characteristics in patients with RE and no-RE showed significant differences regarding age at diagnosis, the site and depth of the tumor, the grade and the histotype and initial and final margins after RE. Most of the patients with RE (71%) were reoperated at one reference center.

### 3.2. Impact of RE on MFS

#### 3.2.1. Kaplan–Meier Curves

**Figure 2:**
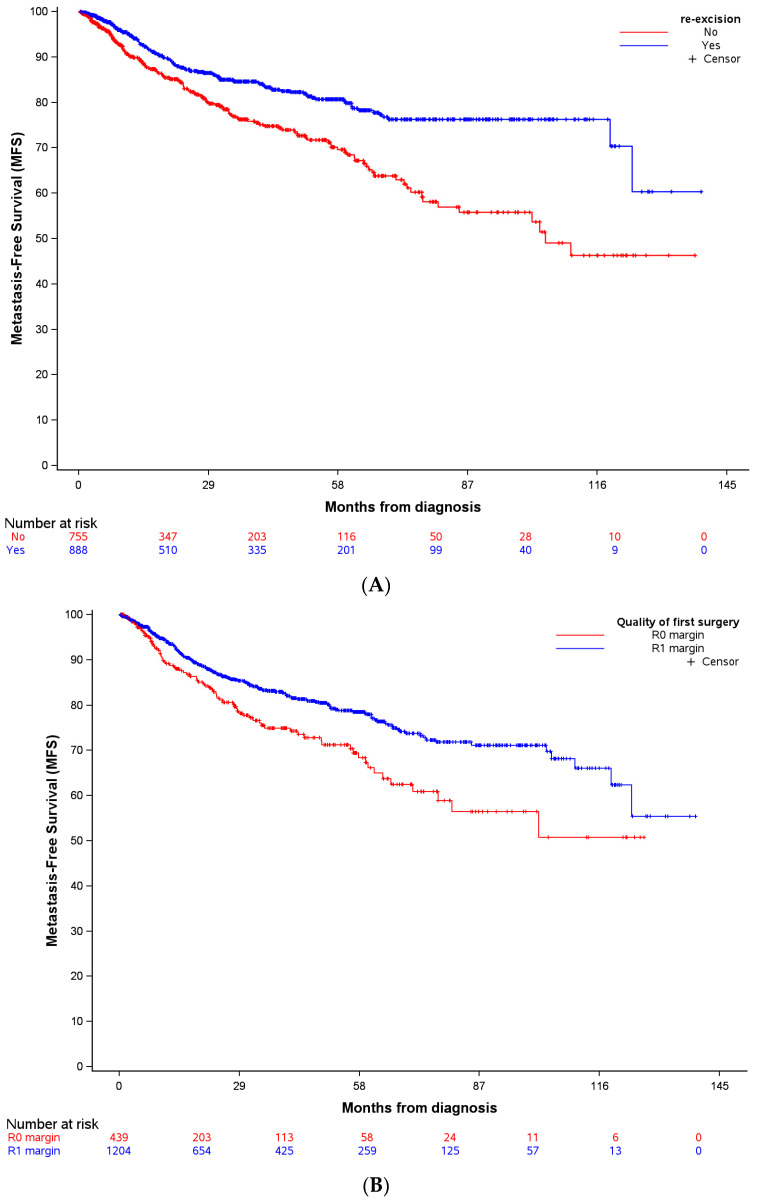

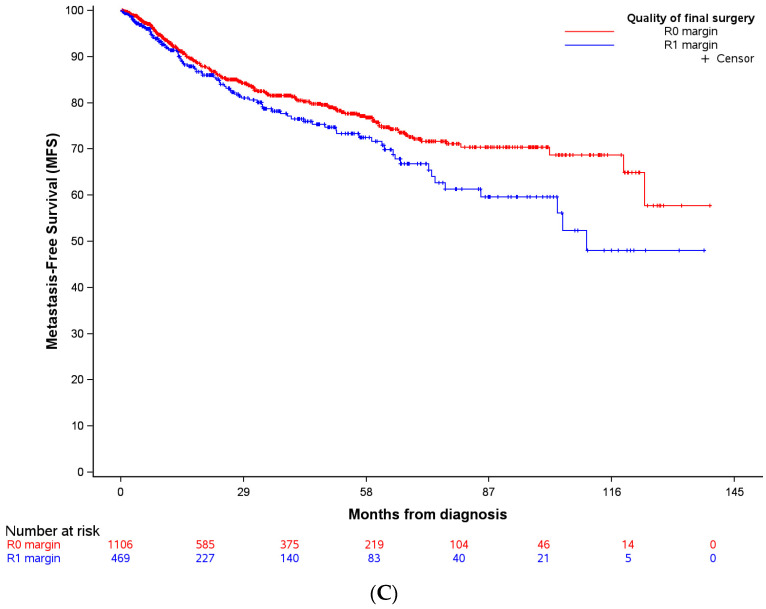
Metastasis-Free Survival (MFS) in patients with complete macroscopic resection outside NETSARC reference centers (out-patients), for whom secondary resection (RE) was performed or not (No-RE) (**A**), according to the quality of the first surgery (**B**), and the quality of the final surgery (**C**). Invalid time, censoring, or strata values deleted: (**A**) n_observations deleted_ = 49; (**B**) n_observations deleted_ = 49; (**C**) n_observations deleted_ = 117.

MFS in patients with UE performed outside NETSARC centers, with RE or no-RE are presented in Figure 2A, according to the quality of the resection based on margin status assessment at first surgical resection (Figure 2B) and at final surgery (Figure 2C). MFS was significantly better in patients with margins qualified as R1 than in patients with margins qualified as R0 following the first surgery (*p* = 0.0016, Figure 2B). Conversely, MFS was significantly improved in patients with final clear margins (R0 margins after RE or R0 initial margins at first surgery in patients not re-operated), compared to final R1 margins (*p* = 0.0388; Figure 2C).

#### 3.2.2. Univariate Analysis

In univariate analysis, RE was associated with an improved MFS (HR 0.57, 95% CI 0.45–0.73), *p* < 0.0001). MFS was also significantly correlated with age at diagnosis (*p* < 0.0001), tumor size (*p* < 0.0001), tumor depth (*p* = 0.0017), tumor grade (*p* < 0.0001), and with histology for myxofibrosarcoma (*p* = 0.0334) (Table 2).

#### 3.2.3. Multivariate Analysis

In the multivariate analysis, age, tumor size and tumor grade remained associated with MFS; RE was associated with better MFS in the full model at the level of 10% (Table 2). The significance increased to the 5% level (HR 0.7, 95% CI 0.53–0.93; *p* = 0.0132) in the final model, after excluding from the model the highly correlated variables initial and final margins (Table 2).

### 3.3. Sub-Group Analysis of Impact of RE on Patients MFS

**Figure 3:**
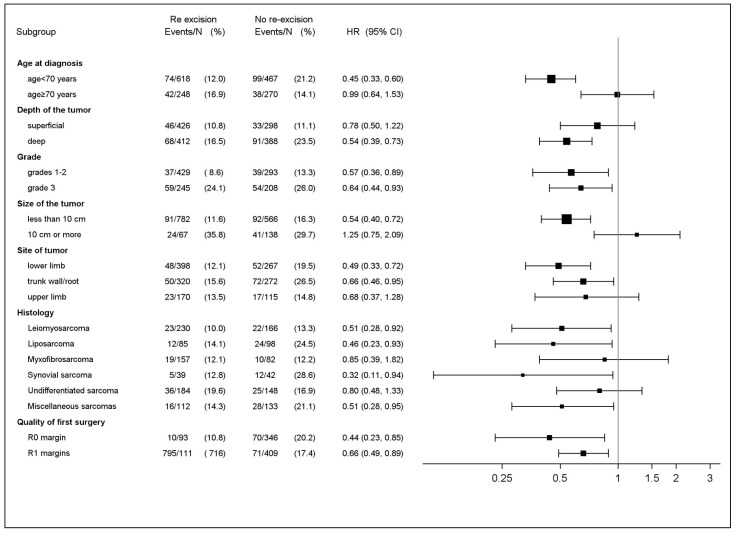
Subgroup analysis and patient Metastasis-Free Survival. (unadjusted hazard ratios (HR); upper CI limit below 1 favors secondary resection (RE) and lower CI limit above 1 favors no secondary resection (No-RE) (*n* = 1692).

We performed a sub-group analysis to identify patient subgroups who may benefit from RE. RE was associated with significantly better MFS in most of the subgroups studied (Figure 3) and interestingly, in all margins initially qualified as either R1 and R0 at first surgery. Nevertheless, this association was not significant in patients older than 70 years, or with tumors larger than 10 cm, sub-fascial tumors, upper-limb tumors and myxofibrosarcoma or with undifferentiated histotype.

In addition, overall survival and cumulative incidence of local recurrence in patients with macroscopic complete resection performed outside NETSARC reference centers (out-patients) with secondary resection (RE) or not (No-RE) are presented in Supplementary Data S1. Univariate and multivariate analyses for overall survival, and local relapse free survival, in out-patients with complete macroscopic margins are presented in Supplementary Data S2 and S3.

## 4. Discussion

This series provided results from the largest cohort of patients with UE performed outside of a reference center, and reports on the MFS of patients with RE and patients with no-RE after referral. Our study shows improved MFS with RE in patients with macroscopically complete UE of a limb or trunk STS performed outside of a NETSARC refer when re-excision was part of adjuvant treatment, regardless of the initial margin status. RE also improved OS and LRFS. Most patient subgroups benefit from RE, except patients older than 70 years and patients with tumors larger than 10 cm. No significant benefit was shown either in myxofibrosarcoma and undifferentiated pleomorphic sarcomas.

After UE of a STS of limb or trunk, RE is generally recommended [3,9,10]. It is generally acknowledged that RE improves local control from 31% to 72%, limiting the high rate of residual disease in tumor-bed resection [5,11,13,14,16,18,19,28]. In addition, improved LRFS is reported in patients with RE as compared to patients in whom no RE is proposed [12,15,17,20]. The present study confirmed that a strategy of systematic RE in patients with STS and macroscopically complete UE performed out of a reference center improves local control, and this is consistent with previous studies. All the studies that directly compare patients with UE, with RE or with no-RE, as shown in the present study, support the concept of systematic RE to improve local control.

The improved MFS after macroscopic complete UE reported in the present study, is less agreed upon. Some authors proposed to postpone the RE at the time of local recurrence if occurring [12,21,22]. Several considerations support this option. First of all, re-excision leads to greater morbidity, and need more complex soft tissue reconstruction and plastic surgery than primary surgery [8,27,30,31,32,33,34]. Second, the impact of residual disease on MFS and survival, occurring in up to 72% of the cases in the re-excision tumor-bed, is still unclear.

Most studies reported that the presence of recurrence is associated with a lower MFS and/or DSS [5,6,17,20,23,24,25,26,27], justifying systematic RE. Traveek et al. [30] reported that recurrences occur in only 13% of the cases and RE-associated morbidity questioned the utility of systematic re-operation. Other authors consider that RE is not associated with improved LRFS [28], MFS, and DSS [22,28]. Finally, some studies report no association between LR and OS [21], or between final margin status and better OS and/or disease metastatic control [21,22,31]. However, the impact of margins was explored in heterogeneous groups of patients, which included patients with first operation performed within a reference center and patients referred after UE.

Finally, all but one of the studies [12] supporting that postponing RE after macroscopic complete UE of limb or trunk STS as an option, rely on indirect evidence, and did not directly explore the outcome of all patients referred and RE and no-RE in patients either referred or not referred.

Only four studies directly compared patients with UE and re-operated and patients with UE not re-operated; after adjustment in a multivariate analysis, Zagars et al. reported better OS and metastatic-free rate in patients with macroscopically complete UE who were re-operated [20]. Nakamura et al. also reported an association between re-operation and better MFS in patients with small (<5 cm) high grade tumors [17]. Our national group using the large multicentric database NETSARC+ reported a better OS and improved recurrence-free survival in patients re-operated after a UE, qualified as R1 [15]. Decanter et al. did not report significantly different OS and MFS in patients with UE re-operated and patients with UE not re-operated, based on data from a subgroup of patients from the national database [12]. These seemingly contradictory conclusions may result from a lack of power related to small sample sizes, and inclusion of some patients with no life-threatening disease (dermatofibrosarcoma protuberans, atypical lipomatous tumor, and desmoid tumor) and limited impact of RE in a majority of tumors after supposed R0 resection. The present study provides direct comparison of patients with UE and RE and patients with UE and no-RE, and reported better MFS in patients reoperated after UE conducted outside a reference center, consistently with three of the four aforementioned studies.

The present study does not allow us to conclude that all patients with macroscopically complete UE initially operated outside reference centers should be re-operated. Better identification of subgroups of patients for whom RE should be recommended, or conversely discouraged, is required. In our subgroup analysis, RE was not associated with better MFS for large tumor (≥10 cm) and in older patients (≥70 years), and no significant benefit in MFS cases was reported in patients with myxofibrosarcoma or undifferentiated pleiomorphic sarcomas. Subgroup analysis is scarce in literature. Nakamura et al. 2020 [17] reported a better OS and lower disease metastatic rate in patients re-operated after an UE of a small (<5 cm) high grade sarcoma. The poorer oncologic outcome in patients with UE not re-operated reported by Takemori et al. [32], was more significant in patients older than 61 years old and in patients with larger tumors (>2.9 cm). Traub et al. [27] explored the oncologic outcome of patients with UE and re-excised, with American Joint Committee on Cancer stage III tumors (≥5 cm, deep, high grade). It is therefore impossible to draw any conclusion from the literature, and clinical groups that might benefit from RE still need to be further defined. Moreover, exploration in patients with good prognosis (i.e., small, superficial and low-grade tumors) who are candidates for a “wait and watch” policy was limited by the reduced sample size and nuber of events (disease-related death). Further studies need to focus on this specific point. So far, RE has to be discussed for all patients after macroscopic complete UE resection outside of a NETSARC reference center.

The present univariate analysis showed that final clear margins (margin status qualified at first operation in patients not re-operated or final margin status after re-operation) are associated with a better MFS, indirectly supporting that clear margin status after UE is critical. This position is consistent with Potter et al. [33] and O’Donnel et al. [34] who considered that R1 margins assessed in patients firstly operated in a reference center could be a biomarker of disease aggressiveness. Other authors did not find any association between margins and metastasis occurrence [21,31,35,36]. Nevertheless, these studies included patients referred and re-operated and patients first operated in a reference center. Finally, only Zagars et al. [20] and Decanter et al. [12] directly compared the impact of margins of all UE (reoperated and not reoperated) similarly to our current analyses, and reported poorer DMFS, DSS in patients with positive or uncertain margin status [20], or any difference on oncologic outcome [12].

Surprisingly, in univariate analysis, outcome in terms of MFS was significantly better in patients with resection qualified as R1 at the first surgery performed out of reference centers, compared to patients with R0 resection. However, surgery qualified as R0 and R1 in this context should be considered with caution. Decision to re-operate a patient referred to a reference center after UE relies on patient general and local assessment, examination of scar track, MRI imaging, careful review of pathologic and operative reports, and MDTB discussion. Re-operation occurred in 913 (53.9%) patients out of the 1692 patients of the whole cohort, including 95 (21.1%) among the 450 patients with initially qualified R0 margins and 818 (65.9%) among the 1242 patients with initial R1 margins. This rate of re-operation is performed at the discretion of local medical teams, and scarce results from the literature reported rates ranging from 44% to 87% [5,15,20,25,37]. The poor prognosis of initial R0 margins raises the issue of appropriate assessment of margin status at first surgery performed out of a reference center and urges us to consider as Noria et al., that no factor currently allows us to predict the presence of microscopic disease, thus all patients with macroscopic complete UE might be eligible for RE and not only patients with first surgery qualified as R1 patients. In addition, any initially qualified R0 resection may provide a falsely reassuring effect and could therefore set patients aside from adjuvant therapy or preclude referral to specialized center. Recently, a predefined surgical plan including a close collaboration between the surgical oncology and the plastic surgery, as currently considered in reference sarcoma centers, should help to further improve results and achieve the most adequate wide oncological resection with acceptable morbidity [38].

There are several limitations in the current study. Firstly, despite a prospective and nationwide data collection, this multicenter retrospective design results in potential selection biases that may affect results: RE decision and to what extent the bed tumor should be re-excised is a critical issue particularly complex to track retrospectively; decisions to perform RE are not based on similar considerations for all patients and all teams. The large sample size, and the current guidelines shared between centers may reduce, but not completely erase this bias. Secondly, we can not exclude that some patients failed to be referred to reference centers by clinicians, or to be registered by pathologista and ultimately were missed. Nevertheless, the nationwide incidence of STS suggests that NETSARC network established a close to exhaustive nationwide data collection from 2013 [2]. Finally, as aforementioned, RE impact on MFS, OS, and LRFS, actually implies considering the complete adjuvant treatment strategy and surveillance modalities associated with the RE process, which were not captured in the present work. Nevertheless, a small and controversial impact of chemotherapy on oncologic outcome is reported in the literature, and radiotherapy was considered not to impact MFS, the primary objective of this study. RE results must be cautiously interpreted in the light of these consensus statements on adjuvant therapy [3,10]. Finally, we relied on multivariate analysis to adjust for observable selection bias. A propensity score method confirming significant impact of RE on MFS has also been used to control the selection bias in accordance with traditional regression for eliminating the bias on observed variables as reported in literature reviews (Supplementary Data S4). However, none of these methods consider the bias due to unobserved variables, i.e., not collected in the study [39].

## 5. Conclusions

For any patient with macroscopic complete unplanned resection of STS of limb or trunk outside of reference center, RE might be considered to improve not only LRFS but also MFS, even in patients with initial margins qualified as R0 margins outside reference centers. Patients older than 70 years and with tumors greater than 10 cm are less likely to benefit from systematic RE.

## Figures and Tables

**Table 1:** Characteristics of patients with first resection performed outside NETSARC reference centers (*n* = 1692).

	Re Excision				Total		Test
	No		Yes				
	N = 779		N = 913		N = 1692		
**Gender**							Chi-2 *p* = 0.092
Female	374	(48.0%)	401	(43.9%)	775	(45.8%)	
Male	405	(52.0%)	512	(56.1%)	917	(54.2%)	
**Age at diagnosis**							Kruskal-Wallis *p* = 0.010
Mean (std)	59.85 (20.39)		57.79 (18.61)		58.74 (19.47)		
**Site of tumor**							Chi-2 *p* ≤ 0.001
Trunk/root member	384	(49.3%)	329	(36.0%)	713	(42.1%)	
Lower limb	278	(35.7%)	411	(45.0%)	689	(40.7%)	
Upper limb	117	(15.0%)	173	(18.9%)	290	(17.1%)	
**Size of the tumor (mm)**							Kruskal-Wallis *p* ≤ 0.001
Mean (std)	63.00 (53.22)		45.97 (38.72)		53.70 (46.63)		
**Depth of the tumor**							Chi-2 *p* = 0.002
Missing data	77		56		133		
Deep (sub-facia)	400	(57.0%)	422	(49.2%)	822	(52.7%)	
Superficial (sus-facia)	302	(43.0%)	435	(50.8%)	737	(47.3%)	
**Histology**							Chi-2 *p* ≤ 0.001
Leiomyosarcoma	170	(21.8%)	237	(26.0%)	407	(24.1%)	
Liposarcoma	106	(13.6%)	88	(9.6%)	194	(11.5%)	
Myxofibrosarcoma	83	(10.7%)	161	(17.6%)	244	(14.4%)	
Synovial sarcoma	43	(5.5%)	41	(4.5%)	84	(5.0%)	
Undifferentiated sarcoma	152	(19.5%)	190	(20.8%)	342	(20.2%)	
Miscellaneous sarcomas	138	(17.7%)	112	(12.3%)	250	(14.8%)	
Others ^¥^	87	(11.2%)	84	(9.2%)	171	(10.1%)	
**Grade of the tumor**							Chi-2 *p* ≤ 0.001
Missing data	64		53		117		
Grade 1	114	(15.9%)	146	(17.0%)	260	(16.5%)	
Grade 2	189	(26.4%)	295	(34.3%)	484	(30.7%)	
Grade 3	212	(29.7%)	249	(29.0%)	461	(29.3%)	
Non gradable	200	(28.0%)	170	(19.8%)	370	(23.5%)	
**Quality of 1st resection**							Chi-2 *p* ≤ 0.001
R0	355	(45.6%)	95	(10.4%)	450	(26.6%)	
R1	424	(54.4%)	818	(89.6%)	1242	(73.4%)	
**2nd surgery**							
Inside NETSARC	-		647	(70.9%)	647	(38.2%)	
Outside NETSARC	-		236	(25.8%)	236	(13.9%)	
Unknown	-		30	(3.3%)	30	(1.8%)	
Quality after re-excision (RE) *							Chi-2 *p* ≤ 0.001
R0	355	(45.6%)	778	(85.2%)	1133	(67.0%)	
R1	424	(54.4%)	61	(6.7%)	485	(28.7%)	
R2	0	(0.0%)	1	(0.1%)	1	(0.1%)	
** Margins not evaluable/not specified**	0	(0.0%)	73	(8.0%)	73	(4.3%)	

* Note: Final margins R0 including initial margins qualified as R0 N = 72; R1 N = 706; Final margins R1 including initial margins qualified as R0 N = 6 and R1 N = 55; Final margins R2 including initial margins qualified as R0 N = 0 and R1 N = 1; Final margins non-evaluable/not specified including initial margins qualified as R0 N = 17 and R1 N = 56. ^¥^ Osteosarcoma, rhabdomyosarcoma, chondrosarcoma, malignant peripheral nerve sheath tumour, suspicion of sarcoma, sarcoma not further specified, other sarcomas.

**Table 2:** Univariate and multivariate analysis for metastasis-free survival (MFS) in patients with complete macroscopic resection achieved at first surgery, outside NETSARC center. HR: Hazard ratio (95% CI); *p* value.

	Unadjusted HR	Adjusted HR Full Model 1	Adjusted HR Full Model 2	Adjusted HR Final Model
Age at diagnosis	1.013 (1.007–1.020); **<0.0001**	1.018 (1.009–1.026); **<0.0001**	1.017 (1.008–1.026); **0.0001**	1.018 (1.009–1.026); **<0.0001**
Gender female (ref: male)	0.825 (0.646–1.053); 0.1227	0.765 (0.579–1.012); 0.0603	0.786 (0.593–1.040); 0.0920	0.773 (0.585–1.021); **0.0698**
Size of the tumor (ref: mm)	1.006 (1.004–1.007); **<0.0001**	1.005 (1.003–1.007); **<0.0001**	1.005 (1.003–1.007); **<0.0001**	1.005 (1.003–1.007); **<0.0001**
Site of tumor (ref: lower limb)				
Trunk/root member	1.264 (0.970–1.646); 0.0829	1.085 (0.806–1.462); 0.5896	1.081 (0.800–1.461); 0.6107	1.088 (0.808–1.465); 0.5799
Upper	0.833 (0.577–1.202); 0.3277	0.861 (0.572–1.296); 0.4738	0.856 (0.568–1.291); 0.4588	0.856 (0.569–1.287); 0.4546
Depth of tumor (ref: superficial)	1.542 (1.177–2.020); **0.0017**	1.304 (0.962–1.767); 0.0875	1.290 (0.950–1.751); 0.1027	1.291 (0.953–1.750); 0.0993
Grade (ref: grades 1–2)				
Grade 3	2.481 (1.854–3.319); **<0.0001**	2.194 (1.571–3.066); **<0.0001**	2.197 (1.566–3.082); **<0.0001**	2.209 (1.581–3.086); **<0.0001**
Not gradable	1.402 (0.992–1.980); 0.0552	1.429 (0.713–2.862); 0.3141	1.370 (0.676–2.775); 0.3826	1.437 (0.716–2.881); 0.3076
Histology (ref: others ^¥^)				
Leiomyosarcoma	0.726 (0.457–1.153); 0.1747	1.003 (0.536–1.879); 0.9918	1.015 (0.540–1.909); 0.9625	0.989 (0.528–1.851); 0.9714
Liposarcoma	0.791 (0.487–1.286); 0.3446	0.541 (0.280–1.044); 0.0670	0.554 (0.284–1.082); 0.0838	0.527 (0.273–1.017); 0.0563
Myxofibrosarcoma	0.575 (0.345–0.957); **0.0334**	0.625 (0.319–1.223); 0.1697	0.599 (0.303–1.182); 0.1395	0.615 (0.314–1.203); 0.1557
Synovial sarcoma	0.867 (0.478–1.573); 0.6390	1.571 (0.741–3.332); 0.2390	1.626 (0.765–3.456); 0.2063	1.559 (0.735–3.307); 0.2473
Undifferentiated sarcoma	0.966 (0.624–1.496); 0.8777	0.801 (0.440–1.457); 0.4667	0.777 (0.424–1.422); 0.4129	0.778 (0.428–1.412); 0.4086
Miscelaneous sarcomas	0.829 (0.521–1.319); 0.4283	0.824 (0.459–1.481); 0.5182	0.887 (0.489–1.607); 0.6919	0.826 (0.460–1.484); 0.5230
Re-excision (RE) (ref: no re-excision (No-RE)	0.571 (0.448–0.729); **<0.0001**	0.750 (0.552–1.019); 0.0656	0.734 (0.526–1.023); 0.0676	0.702 (0.531–0.929); **0.0132**
Quality of first surgery R0 (ref: R1 margin)	1.525 (1.172–1.984); **0.0017**	1.202 (0.862–1.676); 0.2785	––	–
Quality of final surgery R0 (ref: R1 margin)	0.762 (0.589–0.987); **0.0394**	––	1.009 (0.712–1.430); 0.9614	–

^¥^ Osteosarcoma, rhabdomyosarcoma, chondrosarcoma, malignant peripheral nerve sheath tumour, suspicion of sarcoma, sarcoma not further specified, other sarcomas.

## Data Availability

The nationwide database NETSARC (netsarc.org) that support the findings of this study contains information that could compromise privacy of the research participants. The anonymised data sets are available upon reasonable request from the data protection officer of the Léon Bérard cancer center at DPD@lyon.unicancer.fr.

## Supplementary Materials

The following supporting information can be downloaded at: https://www.mdpi.com/article/10.3390/cancers16071365/s1, Supplementary Data S1: Kaplan-Meier of overall survival (OS) (A), and cumulative incidence of local recurrence (B) in patients with macroscopic complete resection performed outside NETSARC reference centers (out-patients) with secondary resection (RE) or not (no secondary resection, No-RE); Supplementary Data S2: Univariate and multivariate analysis for overall survival (OS) of out-patients first operated outside NETSARC center with complete macroscopic margins. Hazard ratio (HR) (95%CI); *p* value; Supplementary Data S3: Univariate and multivariate analysis for local relapse free survival (LRFS) in patients first operated outside NETSARC center (out-patients) with complete macroscopic margins. Hazard ratio (HR) (95%CI); *p* value; Supplementary Data S4: Propensity score matching analysis.
