# Supplementary material for: Improved Metastatic-Free Survival after Systematic Re-Excision Following Complete Macroscopic Unplanned Excision of Limb or Trunk Soft Tissue Sarcoma
Source: Cancers (Basel). 2024 Mar 30;16(7):1365. doi: 10.3390/cancers16071365 (PMC11010862; doi:10.3390/cancers16071365)

Supplementary data

Supplementary data S1. Kaplan-Meier of overall survival (OS)(A), and cumulative incidence of local recurrence (B) in patients with macroscopic complete resection performed outside NETSARC reference centers (out-patients) with secondary resection (RE) or not (no secondary resection, No-RE).

Supplementary data S2. Univariate and multivariate analysis for overall survival (OS) of out-patients first operated outside NETSARC center with complete macroscopic margins. Hazard ratio (HR) (95%CI); p value.

Supplementary data S3. Univariate and multivariate analysis for local relapse free survival (LRFS) in patients first operated outside NETSARC center (out-patients) with complete macroscopic margins. Hazard ratio (HR) (95%CI); p value.

Supplementary data (S4). Propensity score matching analysis.

<u>Supplementary data S1</u>. Kaplan-Meier of overall survival (OS)(A), and cumulative incidence of local recurrence (B) in patients with macroscopic complete resection performed outside NETSARC reference centers (out-patients) with secondary resection (RE) or not (no secondary resection, No-RE).

A

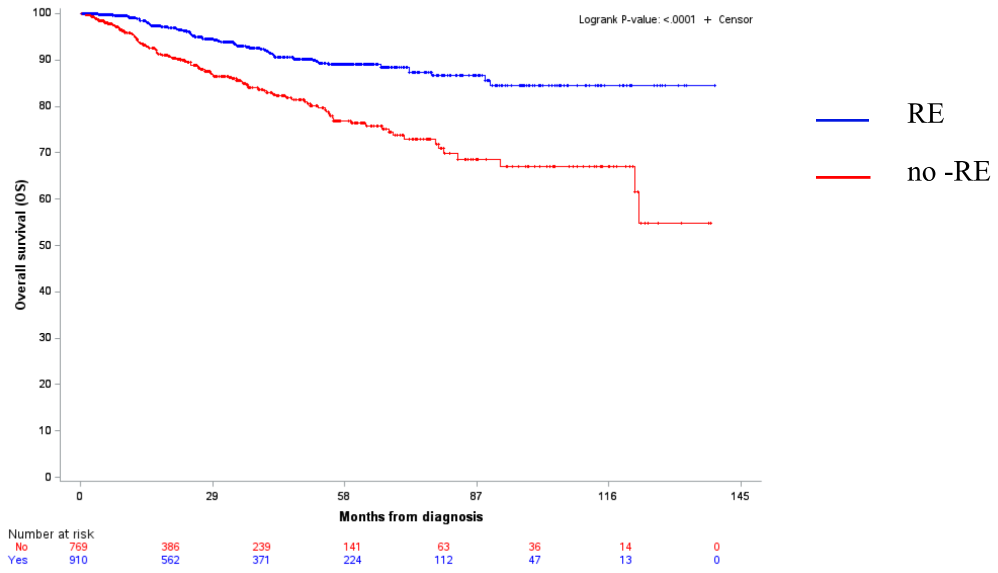

B

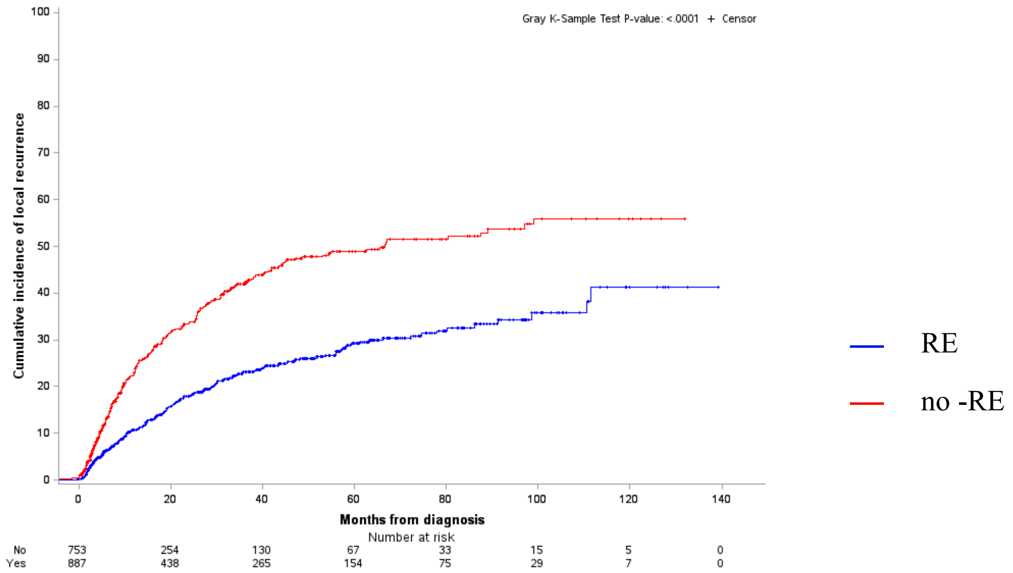

Note. Local relapse free survival (LRFS) was computed from the diagnosis date to the date of last follow-up or the date of first local progression. Overall survival (OS) was defined as the time from the date of diagnosis to the date of the last follow-up or death due to any cause. Competing events to local recurrence were considered in a competing risk approach to estimate LRFS and non-parametric Gray's test were used to estimate and to compare cumulative incidence function between groups. Invalid time, censoring, or strata values A) $n_{observations\ deleted}$ =13; B) $n_{observations\ deleted}$ =57.

Supplementary data S2. Univariate and multivariate analysis for overall survival (OS) of out-patients first operated outside NETSARC center with complete macroscopic margins. Hazard ratio (HR) (95%CI); p value.

	Overall Survival			
	**Unadjusted HR**	**Adjusted HR**	**Adjusted HR**	**Adjusted HR**
**Age at diagnosis**	1.035 (1.025–1.045); **<.0001**	1.036 (1.024–1.048); **<.0001**	1.037 (1.025–1.049); **<.0001**	1.035 (1.023–1.047); **<.0001**
**Gender female (ref: male)**	0.928 (0.682–1.265); 0.6377	0.832 (0.586–1.181); 0.3029	0.846 (0.596–1.203); 0.3524	0.881 (0.617–1.258); 0.4868
**Size of the tumor (mm)**	1.005 (1.003–1.007); **<.0001**	1.004 (1.002–1.007); **0.0005**	1.004 (1.002–1.007); 0.0006	1.004 (1.002–1.007); **0.0006**
**Site of tumor (ref: lower limb)**				
Trunk/root member	1.679 (1.194–2.360); **0.0029**	1.427 (0.979–2.080); 0.0642	1.441 (0.988–2.100); 0.0575	1.530 (1.038–2.254); **0.0317**
Upper	0.838 (0.505–1.388); 0.4918	0.776 (0.447–1.346); 0.3663	0.775 (0.447–1.345); 0.3649	0.841 (0.481–1.470); 0.5440
**Depth of tumor (ref: superficial)**	0.906 (0.657–1.250); 0.5491	0.887 (0.618–1.273); 0.5149	0.875 (0.609–1.257); 0.4688	0.825 (0.572–1.190); 0.3032
**Grade (ref: grades 1–2)**				
Grade 3	2.132 (1.471–3.091); **<.0001**	1.783 (1.170–2.718); **0.0072**	1.775 (1.164–2.708); **0.0077**	1.751 (1.137–2.696); **0.0109**
Not gradable	1.619 (1.072–2.445); 0.0220	0.894 (0.365–2.189); 0.8060	0.885 (0.360–2.173); 0.7893	0.959 (0.379–2.428); 0.9303
**Histology (ref: others[¥])**				
Leiomyosarcoma	0.404 (0.212–0.772); 0.0061	0.435 (0.189–1.004); **0.0512**	0.421 (0.182–0.975); 0.0434	0.463 (0.194–1.101); 0.0814
Liposarcoma	0.545 (0.285–1.041); 0.0661	0.380 (0.163–0.887); **0.0254**	0.358 (0.151–0.848); **0.0195**	0.376 (0.154–0.919); **0.0319**
Myxofibrosarcoma	0.776 (0.433–1.390); 0.3940	0.535 (0.239–1.197); 0.1278	0.514 (0.229–1.156); 0.1076	0.486 (0.207–1.141); 0.0973
Synovial sarcoma	0.520 (0.220–1.230); 0.1363	0.955 (0.333–2.742); 0.9320	0.952 (0.331–2.736); 0.9275	1.065 (0.362–3.128); 0.9093
Undiffferentiated sarcoma	0.981 (0.576–1.672); 0.9447	0.741 (0.361–1.523); 0.4148	0.707 (0.342–1.463); 0.3503	0.735 (0.343–1.574); 0.4279
Miscelaneous sarcomas	0.915 (0.525–1.595); 0.7531	1.276 (0.603–2.700); 0.5238	1.274 (0.602–2.697); 0.5262	1.258 (0.589–2.688); 0.5531
**Re–excision (ref: no)**	0.407 (0.296–0.559); **<.0001**	0.504 (0.354–0.717); **0.0001**	0.470 (0.322–0.687); **<.0001**	0.588 (0.388–0.892); **0.0125**
**Quality of 1st surgery R0 (ref=R1 margin)**	1.373 (0.975–1.933); 0.0692	—	0.816 (0.536–1.243); 0.3443	—
**Quality of final surgery R0 (ref=R1 margin)**	0.485 (0.354–0.665); **<.0001**	—	—	0.701 (0.466–1.054); 0.0880

[¥]Osteosarcoma, rhabdomyosarcoma, chondrosarcoma, malignant peripheral nerve sheath tumour, suspicion of sarcoma, sarcoma not further specified, other sarcomas

Supplementary data S3. Univariate and multivariate analysis for local relapse free survival (LRFS) in patients first operated outside NETSARC center (out-patients) with complete macroscopic margins. Hazard ratio (HR) (95%CI); p value.

	Local relapse free survival			
	**Unadjusted HR**	**Adjusted HR**	**Adjusted HR**	**Adjusted HR**
**Age at diagnosis**	1.015 (1.010-1.021); **<.0001**	1.012 (1.006-1.019); **0.0002**	1.013 (1.006-1.019); **0.0001**	1.013 (1.006-1.019); **0.0001**
**Gender female (ref: male)**	1.026 (0.848-1.240); 0.7946	1.064 (0.862-1.314); 0.5638	1.087 (0.879-1.343); 0.4432	1.089 (0.876-1.354); 0.4429
**Size of the tumor (mm)**	1.004 (1.003-1.006); **<.0001**	1.002 (1.000-1.004); 0.1138	1.002 (1.000-1.004); 0.0685	1.002 (1.000-1.004); 0.1088
**Site of tumor (ref: lower limb)**				
Trunk/root member	1.083 (0.878-1.335); 0.4551	1.146 (0.903-1.455); 0.2618	1.155 (0.911-1.464); 0.2333	1.143 (0.898-1.454); 0.2766
Upper	1.047 (0.803-1.367); 0.7330	1.092 (0.810-1.473); 0.5646	1.088 (0.805-1.469); 0.5839	1.098 (0.808-1.492); 0.5512
**Depth of tumor (ref: superficial)**	1.220 (0.999-1.490); 0.0515	1.229 (0.984-1.536); 0.0690	1.209 (0.968-1.509); 0.0941	1.239 (0.990-1.551); 0.0616
**Grade (ref: grades 1–2)**				
Grade 3	1.303 (1.040-1.633); **0.0213**	1.139 (0.888-1.462); 0.3041	1.149 (0.895-1.476); 0.2751	1.122 (0.867-1.452); 0.3819
No grade assigned	1.107 (0.861-1.422); 0.4271	1.398 (0.841-2.325); 0.1963	1.428 (0.858-2.377); 0.1709	1.473 (0.850-2.553); 0.1675
Histology (ref: others[¥])				
Leiomyosarcoma	0.586 (0.399-0.861); 0.0065	0.868 (0.505-1.492); 0.6086	0.833 (0.483-1.436); 0.5107	0.815 (0.460-1.443); 0.4827
Liposarcoma	0.883 (0.600-1.299); 0.5274	1.028 (0.624-1.694); 0.9138	0.945 (0.569-1.571); 0.8276	0.908 (0.529-1.558); 0.7248
Myxofibrosarcoma	1.173 (0.821-1.678); 0.3808	1.671 (1.010-2.763); **0.0457**	1.559 (0.935-2.600); 0.0885	1.428 (0.826-2.469); 0.2023
Synovial sarcoma	0.653 (0.380-1.120); 0.1214	0.784 (0.361-1.704); 0.5396	0.756 (0.348-1.643); 0.4805	0.666 (0.298-1.488); 0.3214
Undiffferentiated sarcoma	0.963 (0.677-1.369); 0.8320	1.189 (0.729-1.938); 0.4884	1.098 (0.668-1.805); 0.7113	1.024 (0.605-1.735); 0.9283
Miscelaneous sarcomas	0.730 (0.496-1.076); 0.1119	0.727 (0.456-1.158); 0.1794	0.705 (0.444-1.120); 0.1386	0.658 (0.409-1.057); 0.0837
**Re–excision (ref: no)**	0.492 (0.406-0.595); **<.0001**	0.511 (0.410-0.638); **<.0001**	0.443 (0.345-0.570); <.0001	0.706 (0.546-0.913); **0.0079**
**Quality of 1st surgery R0 (ref=R1 margins)**	1.085 (0.877-1.342); 0.4528	---	0.669 (0.501-0.895); **0.0067**	---
**Quality of final surgery R0 (ref=R1 margins)**	0.389 (0.320-0.472); **<.0001**	---		0.499 (0.388-0.644); **<.0001**

[¥]Osteosarcoma, rhabdomyosarcoma, chondrosarcoma, malignant peripheral nerve sheath tumour, suspicion of sarcoma, sarcoma not further specified, other sarcomas

<u>Supplementary data S4.</u> Propensity score matching analysis: after multiple imputations for handling missing covariates, we implemented the inverse probability of treatment weighting (IPTW), a method using the propensity score to control the potential selection bias associated with no categorization. The propensity score was the conditional probability for a patient to be re-excised, conditional on observable characteristics: age, gender, site of tumor, size of tumor, depth of tumor, histology, grade. The resulting graphs confirmed the previously observed better MFS in out NETSARC patients with re-excision (p=0.0006).

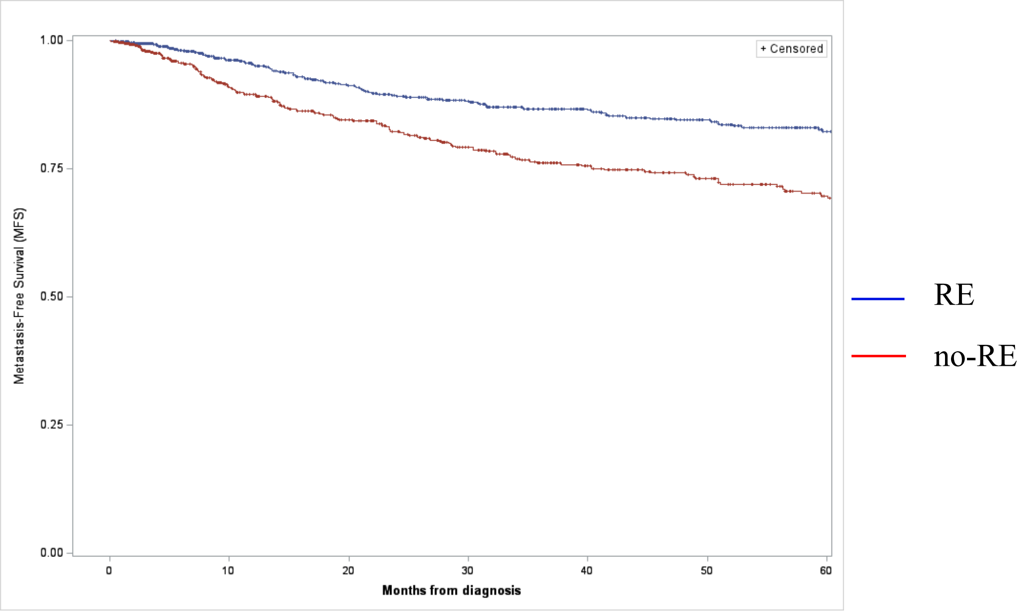

Supplement: Supplementary file 1 [file cancers-16-01365-s001.zip › cancers-2936993-supplementary.pdf]
